# Effects of Exercise-Based Prehabilitation on Functional Capacity in Gastrointestinal Cancer Surgery: A Network Meta-Analysis

**DOI:** 10.3390/jcm15031274

**Published:** 2026-02-05

**Authors:** Minjae Seo, Seokjun Cho, Eunbi Kim, Jooyeol In, Jaesung Lee, Jungmin Lee, Jonghoon Park, Jae-Seok Min

**Affiliations:** 1 Exercise Nutrition and Biochemistry Laboratory, Department of Physical Education, Korea University, 145 Anam-ro, Seongbuk-gu, Seoul 02841, Republic of Korea; zzee97@korea.ac.kr (M.S.); zwx0209@korea.ac.kr (S.C.); eunbi22_kr@korea.ac.kr (E.K.); dlswnduf@korea.ac.kr (J.I.); dlwotjd1506@korea.ac.kr (J.L.); jamielee@korea.ac.kr (J.L.); 2 Department of Surgery, Korea University College of Medicine, Seoul 02841, Republic of Korea; 3 Division of Foregut Surgery, Korea University Anam Hospital, Seoul 02841, Republic of Korea

**Keywords:** gastrointestinal neoplasm, surgery, prehabilitation, meta-analysis, postoperative outcomes

## Abstract

**Objectives**: This study aimed to compare the effects of facility-based and home-based exercise prehabilitation on postoperative functional capacity and postoperative outcomes in patients undergoing gastrointestinal cancer surgery using a network meta-analysis. **Methods**: This systematic review and network meta-analysis included randomized controlled trials of exercise-based prehabilitation in patients undergoing gastrointestinal cancer surgery. Facility-based and home-based exercise interventions were compared using random-effects models to evaluate postoperative functional capacity, overall complications, and length of stay. Certainty of evidence was assessed using the CINeMA framework. **Results**: Nine randomized controlled trials were included. Exercise-based prehabilitation significantly improved postoperative functional capacity compared with control in pairwise meta-analysis (MD = 26.10 m; 95% CI 4.59 to 47.62). In network meta-analysis, facility-based exercise prehabilitation significantly improved postoperative functional capacity compared with control (MD = 24.11 m; 95% CI 2.01 to 46.22), whereas home-based prehabilitation showed no statistically significant effect (MD = 32.12 m; 95% CI −1.70 to 65.93). **Conclusions**: This study suggests that exercise prehabilitation may be an effective strategy for improving or preserving postoperative functional capacity in patients undergoing gastrointestinal cancer surgery, with facility-based exercise prehabilitation showing more consistent and statistically significant effects.

## 1. Introduction

Gastrointestinal cancer refers to malignant tumors arising in the gastrointestinal tract, including the esophagus, stomach, small intestine, and colon, and represents a major cancer type with high incidence and mortality worldwide [1]. In the treatment of gastrointestinal cancer, surgical resection is commonly considered for patients with resectable disease [2]. However, postoperative declines in physical function frequently coincide with complications that extend hospitalization, escalate healthcare costs, and elevate postoperative mortality [3,4].

Prehabilitation, a set of pre-surgical targeted interventions, has recently gained attention as an approach to improve postoperative clinical outcomes. Prehabilitation aims to optimize the physical and psychological status of patients before surgery through exercise, nutritional, and psychological interventions, with the goal of enhancing postoperative functional recovery [5]. Among these components, exercise interventions are consistently included in previous studies and are considered a key element, as they directly influence the physical function and fitness levels of patients. These exercise interventions are often evaluated with the 6-minute walk distance (6MWD), a functional assessment tool that reflects physical capacity. It has been validated as a measure of functional exercise capacity not only in patients with respiratory and cardiovascular diseases, but in patients with cancer as well [6,7].

Recent systematic reviews have reported that exercise-based prehabilitation, depending on the mode of intervention, varies widely in terms of exercise type and delivery setting, and that these differences may influence both intervention effectiveness and clinical applicability [8]. Exercise-based prehabilitation can be broadly classified into facility-based interventions, conducted in healthcare facilities under the guidance of healthcare professionals, and home-based interventions, in which patients perform exercise autonomously at home after initial instruction. Facility-based interventions allow structured monitoring of exercise intensity and adherence and provide individualized feedback through in-person supervision. However, several barriers may negatively affect participation and long-term adherence, including the need for regular hospital visits, limited accessibility, and associated time and financial burdens [9]. In contrast, home-based interventions, while offering greater accessibility, may require substantial motivation and self-regulation for sedentary cancer patients, potentially limiting the maintenance of long-term behavioral change [10].

Previous studies have reported positive outcomes from both approaches. According to a meta-analysis by Kraemer et al., fully facility-based or mixed exercise interventions in patients with colorectal cancer demonstrated significant improvements in quality of life and functional capacity [11]. In addition, studies incorporating facility-based high-intensity exercise within prehabilitation programs reported reductions in the Comprehensive Complication Index and length of hospital stay [12]. Conversely, meta-analyses including home-based interventions have also reported improvements in 6MWD and reductions in postoperative complications following prehabilitation [13]. Yet among systematic reviews and meta-analyses evaluating exercise-based prehabilitation in patients with gastrointestinal cancer, studies directly comparing the relative effects of facility-based and home-based exercise interventions remain limited. Most of these meta-analyses are based on pairwise comparisons, which prevent them from simultaneously analyzing both intervention strategies. Moreover, studies that apply network meta-analysis (NMA) to address this limitation are scarce. Network meta-analysis enables the simultaneous comparison of the relative effects of multiple interventions by integrating direct and indirect evidence, thereby allowing treatment comparisons even when direct comparative evidence is limited [14].

Accordingly, this study aimed to comprehensively evaluate the effects of exercise-based prehabilitation in patients undergoing gastrointestinal cancer surgery using a meta-analysis, and to analyze the relative effects on key clinical outcomes, including postoperative functional outcomes, postoperative complications, and length of stay. Furthermore, this study seeks to identify the most clinically effective prehabilitation strategies for patients with gastrointestinal cancer, and to provide evidence that supports the selection of such interventions in the context of real-world clinical settings.

## 2. Materials and Methods

This study was conducted as a systematic review and network meta-analysis, and the study protocol was prospectively registered in the International Prospective Register of Systematic Reviews (PROSPERO; registration number: CRD420251152758). This guidelines of the Preferred Reporting Items for Systematic Reviews and Meta-analyses extension for Network Meta-analyses (PRISMA-NMA) were followed; the PRISMA-NMA checklist is provided in the Supplementary Material (Table S1).

### 2.1. Eligibility Criteria

This meta-analysis included only randomized controlled trials (RCTs). The study population was restricted to patients who underwent surgery for gastrointestinal cancer. In this review, gastrointestinal cancer was operationally defined as malignancies arising from hollow viscus organs of the gastrointestinal tract. Eligible interventions were defined as studies that implemented exercise-based prehabilitation during the preoperative period. Comparator groups included control groups that received usual care or did not receive structured exercise interventions during the preoperative period. The primary outcome was postoperative functional capacity. Only studies published in English and available as full-text articles were included.

Exercise-based prehabilitation interventions were categorized according to the presence of in-person supervision. Facility-based exercise prehabilitation was defined as interventions conducted in a clinical or institutional setting, with at least one in-person supervised exercise session per week under the direct supervision of a qualified professional. Home-based exercise prehabilitation was defined as interventions conducted primarily in the home setting without any in-person supervision, in which adherence was monitored exclusively through remote methods such as telephone or video-based contact.

### 2.2. Information Sources and Search Strategy

A systematic literature search was conducted using the PubMed, Embase, Web of Science, and Cochrane Library databases. Each database was searched from inception to 2 October 2025. The search strategy was developed based on the core concepts of exercise-based prehabilitation, gastrointestinal cancer, and functional capacity. Relevant keywords for each concept were combined for the search. The detailed search strategies for each database are provided in the Supplementary Material (Table S2).

### 2.3. Literature Screening and Study Selection

To determine which studies should be included, two reviewers independently screened the titles and abstracts of potentially eligible studies. The full texts of the remaining eligible studies were then assessed for inclusion. Disagreements between reviewers were resolved through discussion; when consensus could not be reached, a third reviewer made the final decision. In cases where a single study resulted in multiple publications, the most comprehensive report or the report presenting the primary outcomes was used as the representative publication.

### 2.4. Data Extraction and Outcomes

Two reviewers independently extracted data from the included studies, collecting information on study characteristics and outcome data. Extracted data included study design, participant characteristics, intervention characteristics, and primary and secondary outcome variables.

The primary outcome was defined as postoperative functional capacity and was assessed using the 6MWD. The 6MWD was analyzed as the change from baseline in all included studies. The mean change and corresponding standard deviation (SD) were extracted for each group. When the SD of the change score was not directly reported, it was calculated according to the recommendations of the Cochrane Handbook by assuming a correlation coefficient r of 0.5 between baseline and follow-up measurements [15]. To evaluate the robustness of this assumption, sensitivity analyses were additionally performed using alternative correlation coefficients. When results were reported as medians, interquartile ranges, or 95% confidence intervals, they were converted to means and standard deviations using established methods [15,16]. The prespecified time point for outcome assessment was 4 weeks postoperatively; if data at this time point were unavailable, results from the closest available time point were used.

Secondary outcomes were defined as postoperative outcomes. Overall complications were defined as the occurrence of any complication within 30 days after surgery. Length of stay (LOS) was defined as the duration of hospitalization in days from surgery to discharge. To ensure consistency in the study population across outcomes, secondary outcomes were analyzed using data from the subset of included trials that reported postoperative functional capacity.

### 2.5. Risk of Bias Assessment

The risk of bias of the included RCTs was assessed using the Cochrane Risk of Bias 2 tool [17]. Risk of bias assessment was conducted independently by two reviewers, and any disagreements were resolved through discussion. Risk of Bias was assessed at the outcome level for the primary outcome, and it informed the assessment of certainty of evidence. The results of the risk of bias assessment are presented in the Supplementary Material (Figure S1). Risk of bias and certainty of evidence assessments were conducted for the primary outcome, which defined the study inclusion set and had sufficient network connectivity to allow formal assessment. Secondary outcomes were analyzed within the same trials. This should be considered when interpreting their results.

### 2.6. Data Synthesis and Statistical Analysis

For each outcome, both pairwise meta-analysis and frequentist NMA were performed. Mean difference (MD) was used as the effect measure for continuous outcomes, and risk ratio (RR) was used for binary outcomes. All analyses were conducted using a random-effects model to account for potential clinical and methodological heterogeneity across studies.

Pairwise meta-analyses were first conducted for studies with direct comparisons. NMA, which integrates direct and indirect evidence to estimate relative treatment effects, was then performed to simultaneously estimate the relative effects among all interventions within the network for the same outcomes. The results are presented using network meta-analysis forest plots and league tables. Treatment ranking was assessed using P-scores. Assessment of inconsistency was planned where feasible; global inconsistency was evaluated at the network level, whereas the present network had a limited number of closed loops, which restricted formal evaluation of local inconsistency. Accordingly, information related to inconsistency was summarized where possible in the Supplementary Material. Small-study effects and potential publication bias were explored using funnel plots.

The network structure was visualized using network geometry. In the network geometry, nodes represent intervention types, and edges indicate direct comparisons between interventions. The size of each node is proportional to the number of studies included in the corresponding comparisons.

All statistical analyses were performed using R software (version 4.5.1) [18]. The meta package (version 8.2-1) was used for pairwise meta-analyses, and the netmeta package (version 3.2-0) was used for network meta-analyses [19,20].

### 2.7. Certainty of Evidence

The certainty of evidence was assessed based on the Grading of Recommendations Assessment, Development and Evaluation approach. To account for the specific features of NMA, the Confidence in Network Meta-Analysis (CINeMA) framework was used to evaluate the certainty of evidence [21]. Assessments were conducted across six domains: within-study bias, reporting bias, indirectness, imprecision, heterogeneity, and incoherence. Domain-level judgments were then integrated to determine the overall certainty of evidence for each comparison.

## 3. Results

### 3.1. Study Selection

**Figure 1:**
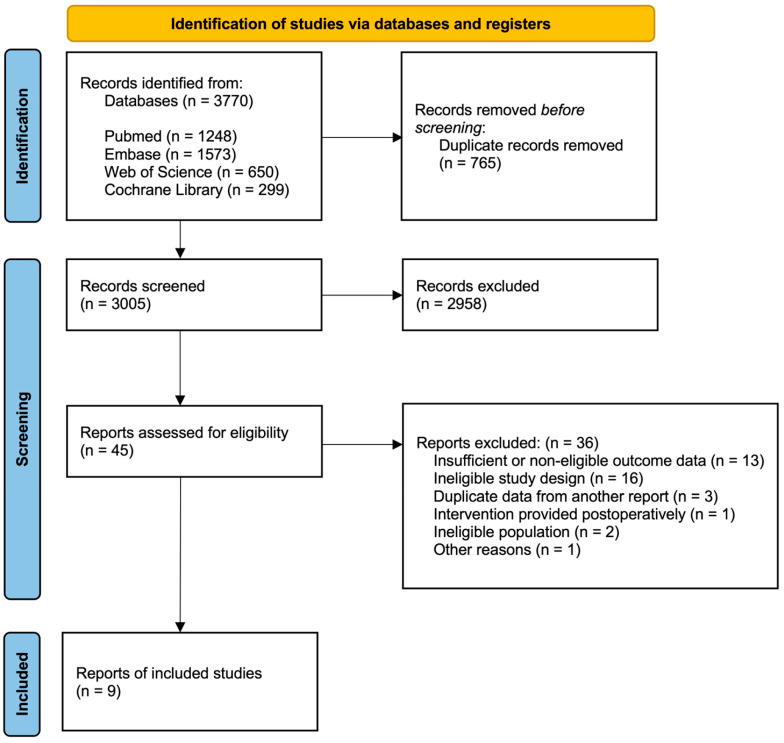
PRISMA 2020 flow diagram of study selection.

A total of 3770 records were identified through database searches. After removal of duplicate records (n = 765), titles and abstracts were screened, and 2958 records were excluded. Full-text articles were subsequently assessed for eligibility, and an additional 36 studies were excluded due to reasons such as study design, timing of the intervention, or ineligible outcome measures. A total of nine RCTs met the inclusion criteria and were included in both the qualitative and quantitative syntheses. The study selection process is presented in the PRISMA 2020 flow diagram (Figure 1).

### 3.2. Study Characteristics

A total of nine RCTs were included in this meta-analysis. The study populations consisted of patients scheduled for surgery for gastrointestinal cancer. Of the included studies, seven focused on patients with colorectal cancer, one included patients with gastric cancer, and one included patients with esophageal and gastric cancer.

In all studies, exercise-based prehabilitation interventions were implemented during the preoperative period, categorized as facility-based or home-based exercise prehabilitation according to the presence of facility-based supervision. The duration of the interventions and the exercise components varied across studies. For all included studies, postoperative functional capacity was assessed using the 6MWD. In addition, some studies also reported postoperative clinical outcomes, including overall complications and LOS. The main characteristics of the included studies are summarized in Table A1.

### 3.3. Risk of Bias, Certainty of Evidence, and Inconsistency

Risk of bias assessments for the primary outcome (6MWD) are presented in Figure S1. In the domain of bias due to deviations from intended interventions, several studies were judged to be at high risk of bias, which contributed to high overall risk of bias judgments in these studies.

**Figure 2:**
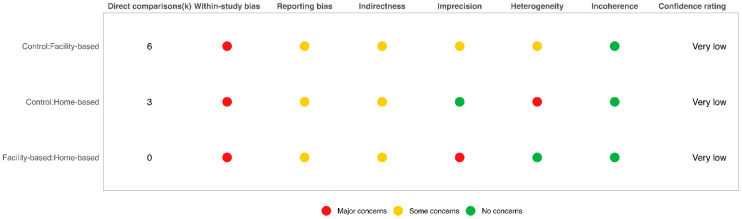
Confidence in network meta-analysis (CINeMA) assessment.

The risk of bias assessments was incorporated into the CINeMA framework as the within-study bias domain. As a result, major concerns were identified in the within-study bias domain. In addition, major concerns were also observed in the domains of heterogeneity and incoherence, which collectively led to a low certainty of evidence for the network meta-analysis results. Detailed domain-level assessments and overall judgments are provided in Figure 2 and Supplementary Table S3.

Consistency across the network was evaluated using a global inconsistency test (Table S4). At the network level, total inconsistency was not statistically significant (Q = 13.67, df = 7, *p* = 0.057), indicating no evidence of overall global inconsistency. However, in design-specific analyses, significant inconsistency was observed in comparisons between control and home-based prehabilitation, whereas no evidence of inconsistency was identified in comparisons between control and facility-based exercise prehabilitation.

### 3.4. Effects of Exercise-Based Prehabilitation

#### 3.4.1. Functional Capacity (6MWD)

**Figure 3:**
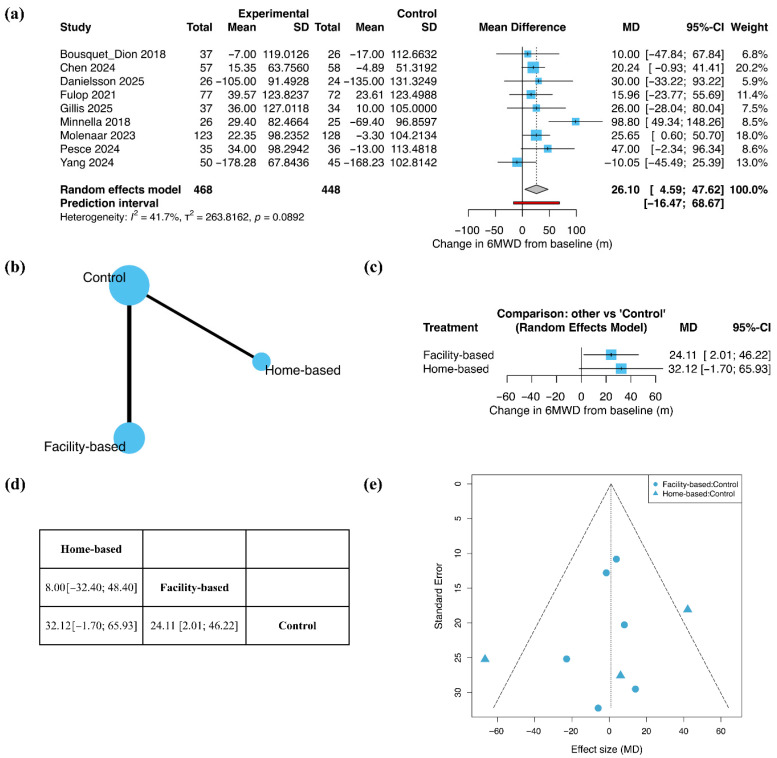
Functional capacity (6MWD): (**a**) Pairwise meta-analysis forest plot; (**b**) Network geometry; (**c**) Network meta-analysis forest plot; (**d**) League table; (**e**) Funnel plot. In forest plots, squares represent individual study estimates and diamonds represent pooled effect estimates. Bold text highlights the summary estimate from the random-effects model. The red rectangle denotes the prediction interval.

Functional capacity was assessed using changes in the 6MWD, expressed as MD (Figure 3). In the pairwise meta-analysis including all nine studies, exercise-based prehabilitation was associated with a significant improvement in 6MWD compared with control (MD = 26.10 m; 95% CI 4.59 to 47.62; Figure 3a), with moderate heterogeneity across studies (I^2^ = 41.7%).

The treatment network comprised six studies evaluating facility-based prehabilitation and three evaluating home-based prehabilitation, with most direct comparison conducted against control (Figure 3b). In the network meta-analysis, facility-based exercise prehabilitation resulted in a significant improvement in 6MWD compared with control (MD = 24.11 m; 95% CI 2.01 to 46.22). Home-based exercise prehabilitation showed a tendency toward improvement; however, this effect did not statistically significance (MD = 32.12 m; 95% CI −1.70 to 65.93; Figure 3c). In the league table summarizing relative treatment effects, no clear difference was observed between facility-based and home-based exercise prehabilitation (MD = 8.00 m; 95% CI −32.40 to 48.40; Figure 3d). Sensitivity analyses using alternative assumptions for the pre–post correlation coefficient showed that the magnitude and statistical significance of the effects varied according to the assumed correlation. When r was set to 0.3, pairwise meta-analysis showed a significant improvement compared with control (MD = 24.92 m; 95% CI 4.76 to 45.07), and network meta-analysis showed significant effects for facility-based exercise prehabilitation (MD = 23.79 m; 95% CI 2.41 to 45.17), whereas the effect for home-based prehabilitation was not statistically significant (MD = 29.77 m; 95% CI −4.89 to 64.43). When r was set to 0.7, similar results were observed in pairwise meta-analysis (MD = 27.34 m; 95% CI 4.89 to 49.80), with facility-based (MD = 24.40 m; 95% CI 2.17 to 46.63) and home-based exercise prehabilitation (MD = 34.50 m; 95% CI 1.84 to 67.17) showing significant effects compared with control (Figure S2). Funnel plot inspection suggested an overall symmetric distribution of effect estimates, although interpretation is limited by the small number of included studies (Figure 3e).

#### 3.4.2. Overall Complications

**Figure 4:**
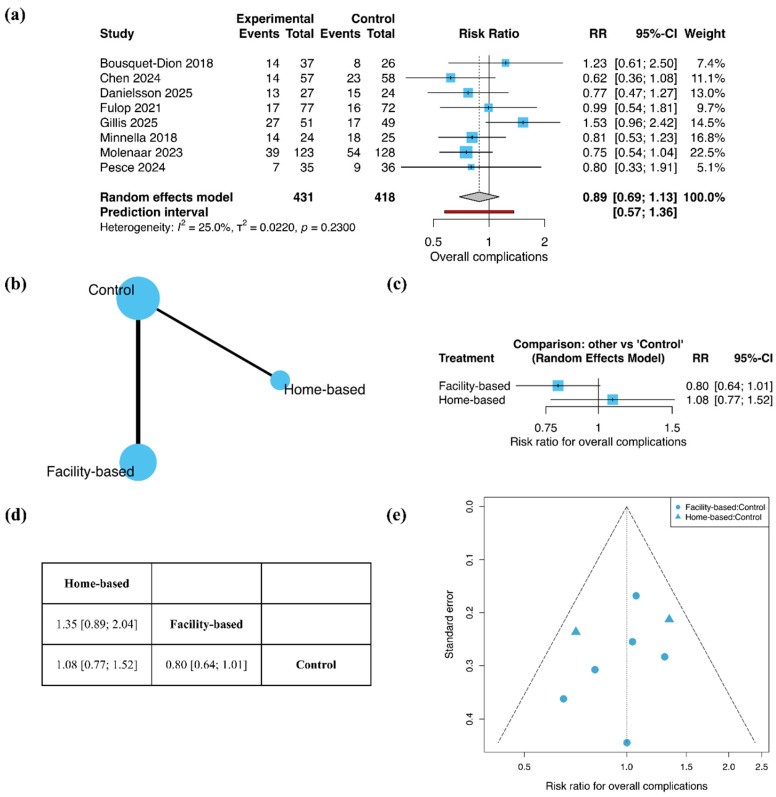
Overall complications: (**a**) Pairwise meta-analysis forest plot; (**b**) Network geometry; (**c**) Network meta-analysis forest plot; (**d**) League table; (**e**) Funnel plot. In forest plots, squares represent individual study estimates and diamonds represent pooled effect estimates. Bold text highlights the summary estimate from the random-effects model. The red rectangle denotes the prediction interval.

Overall complications were analyzed using RR (Figure 4). In the pairwise meta-analysis including eight studies, exercise-based prehabilitation was not associated with a significant reduction in postoperative overall complications compared with control (RR = 0.89; 95% CI 0.69 to 1.13; Figure 4a), with low between-study heterogeneity (I^2^ = 25.0%).

The treatment network included six studies of facility-based prehabilitation and two studies of home-based prehabilitation (Figure 4b). In the network meta-analysis, facility-based exercise prehabilitation showed a non-significant trend toward a lower risk of overall complications compared with control (RR = 0.80; 95% CI 0.64 to 1.01). Home-based exercise prehabilitation was not associated with a reduction in complications (RR = 1.08; 95% CI 0.77 to 1.52; Figure 4c). No clear difference was identified between facility-based and home-based exercise prehabilitation in the league table analysis (RR = 1.35; 95% CI 0.89 to 2.04; Figure 4d). Funnel plot inspection did not indicate substantial asymmetry (Figure 4e).

#### 3.4.3. Length of Stay

**Figure 5:**
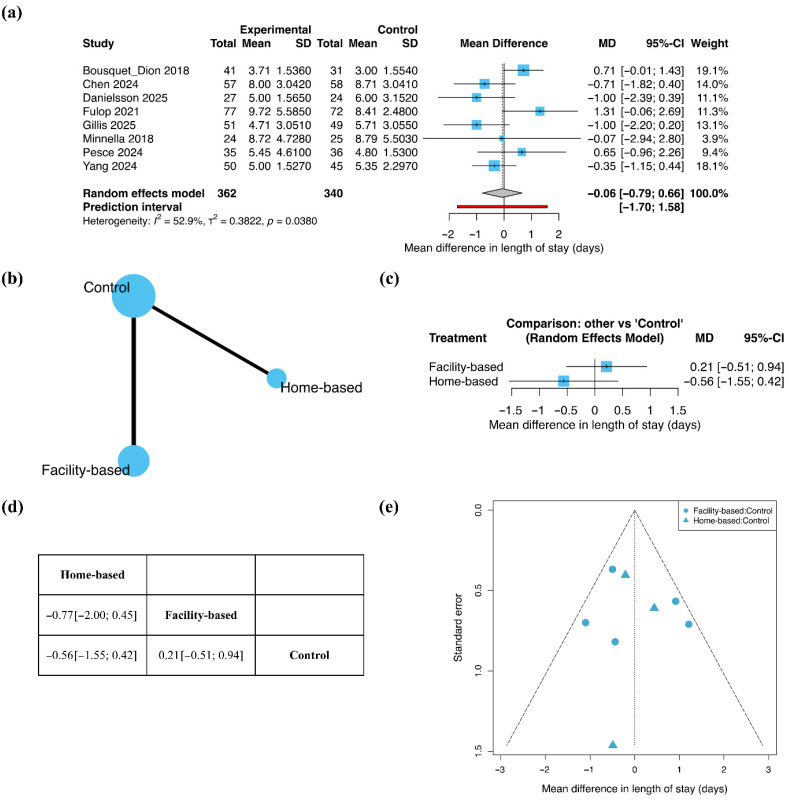
Length of stay: (**a**) Pairwise meta-analysis forest plot; (**b**) Network geometry; (**c**) Network meta-analysis forest plot; (**d**) League table; (**e**) Funnel plot. In forest plots, squares represent individual study estimates and diamonds represent pooled effect estimates. Bold text highlights the summary estimate from the random-effects model. The red rectangle denotes the prediction interval.

Length of stay was analyzed using MD (Figure 5). In the pairwise meta-analysis including eight studies, exercise-based prehabilitation did not result in a significant difference in LOS compared with control (MD = −0.06; 95% CI −0.79 to 0.66). Moderate heterogeneity was observed (I^2^ = 52.9%).

The network included five studies evaluating facility-based exercise prehabilitation and three studies evaluating home-based exercise prehabilitation (Figure 5b). In the NMA, neither facility-based exercise prehabilitation (MD = 0.21; 95% CI −0.51 to 0.94) nor home-based exercise prehabilitation (MD = −0.56; 95% CI −1.55 to 0.42) demonstrated a significant reduction in LOS compared with control (Figure 5c). Consistent with these findings, the league table showed no clear difference between facility-based and home-based exercise prehabilitation (MD = −0.77; 95% CI −2.00 to 0.45; Figure 5d). Funnel plot inspection indicated an overall symmetric distribution of effect estimates (Figure 5e).

## 4. Discussion

This study synthesized evidence from studies involving patients undergoing gastrointestinal cancer surgery to evaluate the effects of exercise-based prehabilitation and to compare the effects of facility-based and home-based exercise prehabilitation. Although no RCTs directly compared facility-based and home-based exercise prehabilitation, indirect comparison was made possible through a network meta-analysis approach. The results of this study showed that exercise prehabilitation significantly improved postoperative 6MWD compared with control conditions, indicating that exercise prehabilitation is an effective strategy for preventing postoperative declines in functional capacity in patients undergoing gastrointestinal cancer surgery. Meanwhile, facility-based exercise prehabilitation demonstrated consistent and statistically significant improvements in 6MWD. Although home-based exercise prehabilitation showed improvements in the same direction, these were accompanied by substantial uncertainty and did not achieve statistical significance. This study also examined the effects of exercise prehabilitation on hard clinical endpoints, including overall complications and length of stay. No significant improvements were observed in either outcome following exercise prehabilitation, and no statistically significant difference was found between facility-based and home-based approaches.

Despite heterogeneity among the included studies, this meta-analysis demonstrated that exercise-based prehabilitation improved postoperative 6MWD in patients undergoing gastrointestinal cancer surgery, aligning with previous evidence that moderate-intensity aerobic exercise, alone or in combination with resistance training, enhances functional capacity in cancer populations [22,23]. The 6MWD is a validated and practical clinical measure that objectively reflects the functional capacity of patients [7,24]. Therefore, exercise prehabilitation can be considered an effective strategy for improving postoperative functional capacity in this patient population. Results from the network meta-analysis indicated that facility-based exercise prehabilitation consistently and significantly improved postoperative 6MWD. The presence of facility-based supervision may have enhanced patient confidence in exercising and increased motivation, leading to favorable changes in actual functional capacity [25]. In patients undergoing abdominal surgery, including gastrointestinal cancer surgery, an anchor-based analysis has suggested that a change of approximately 14 m in the 6MWD represents the minimal clinically important difference, with values approaching 20 m reflecting a clearly clinically meaningful improvement [26]. Although home-based exercise prehabilitation appeared to show effects comparable to or even larger than those of facility-based programs, the wide confidence intervals and lack of consistency across studies indicated considerable uncertainty. This may reflect a trade-off between improved accessibility and reduced social barriers on the one hand, and the absence of structured monitoring on the other, which may have limited adherence and increased uncertainty [27,28].

In contrast to the observed effects on postoperative 6MWD, hard clinical endpoints such as overall complications and LOS were not significantly affected by exercise prehabilitation. This suggests that improvements in functional capacity achieved through exercise prehabilitation do not necessarily translate into improvements in hard clinical outcomes. Regarding overall complications, the pooled risk ratio was below 1; however, the confidence interval encompassed the null value, and the wide prediction interval indicated a marked variability in treatment effects. These findings suggest that any potential benefit was not consistently observed and may have varied across clinical settings, thereby limiting the generalizability of the overall estimate. This is likely because overall postoperative complications in patients undergoing cancer surgery are influenced by multiple factors beyond exercise interventions, including surgical complexity, cancer stage, and perioperative risk profiles, which collectively determine the magnitude of surgical stress and the vulnerability of the patient to postoperative complications and prolonged hospitalization [29,30]. When examined separately, facility-based exercise prehabilitation was associated with a directionally favorable but non-significant reduction in overall complications compared with control (95% CI 0.64 to 1.01), whereas no clear or consistent pattern was observed for home-based exercise prehabilitation. One important limitation of the present analysis is that overall complications were treated as a binary outcome, which precludes consideration of complication severity. Based on a previous study, the presence of major postoperative complications, rather than minor complications, was more closely associated with postoperative recurrence-free survival and overall survival, suggesting that the severity of postoperative complications may be more important than simply the number of complications [31]. Furthermore, although not limited to exercise alone, a previous study reported that multimodal prehabilitation including exercise in patients with gastrointestinal cancers was associated with an approximately 16% reduction in major postoperative complications (Clavien-Dindo grade III–IV) [32]. Future studies should therefore distinguish between major and minor complications to more precisely delineate the clinical impact of exercise prehabilitation.

In the network meta-analysis, exercise prehabilitation showed a negligible overall effect on postoperative LOS after gastrointestinal cancer surgery. Moreover, neither facility-based nor home-based exercise prehabilitation demonstrated a statistically significant reduction in LOS compared with usual care. This may be because LOS in gastrointestinal surgery patients is not determined solely by their physiological recovery, but rather by the combined influence of patient-related factors, surgical complexity, and healthcare system factors, such as unclear discharge criteria [33]. A previous study demonstrated that early discharge could be safely achievable by applying simplified discharge criteria to patients receiving the Enhanced Recovery After Surgery protocol, suggesting that factors beyond the physiological or functional status of patients may play a substantial role in determining LOS [34]. In addition, most included studies implemented exercise prehabilitation alongside ERAS pathways, which may have produced a ceiling effect, leaving limited room for further improvement in postoperative outcomes [35,36]. Therefore, future studies should more carefully evaluate the effect of exercise prehabilitation on LOS in patients undergoing gastrointestinal cancer surgery, while accounting for healthcare system-related factors that may influence LOS, as well as a possible ceiling effect due to concurrent ERAS pathways.

This study has several limitations. First, no RCTs directly compared facility-based and home-based exercise prehabilitation. Although this limitation was addressed through the use of network meta-analysis, the findings are based on indirect comparisons, which restrict the ability to draw conclusions regarding the causal superiority of facility-based exercise prehabilitation over home-based approaches. Accordingly, the results should be interpreted with caution. In addition, the CINeMA assessment indicated that within-study bias persisted in the included studies, and substantial heterogeneity was observed due to variations in intervention protocols, underscoring the need for future well-designed RCTs. Furthermore, differences in inclusion and exclusion criteria across studies, as well as the inclusion of some surgical patients without cancer diagnoses, may have obscured potential effects on overall complications and length of hospital stay. Although some home-based exercise prehabilitation programs included extensive remote monitoring, sensitivity analyses excluding these trials could not be performed. Such exclusions would have resulted in a severely disconnected network, leaving an insufficient number of studies in the home-based category to support stable and meaningful network meta-analytic estimates. This limitation should be considered in the context of the study findings. Nevertheless, the direction of effect for the facility-based category was consistent across studies, lending support to the primary conclusion regarding facility-based programs despite this limitation. Finally, the limited number of included trials precluded subgroup analyses and meta-regression. As a result, potential effect modification by patient characteristics, cancer type, or intervention duration could not be formally assessed, further limiting interpretation of the findings.

## 5. Conclusions

This study suggests that exercise prehabilitation may represent a promising strategy for improving or preserving postoperative functional capacity in patients undergoing gastrointestinal cancer surgery, with facility-based exercise prehabilitation showing more consistent and statistically significant effects. In contrast, although home-based exercise prehabilitation did not achieve statistical significance, its effect sizes were comparable to or potentially larger than those observed with facility-based programs. These findings indicate that incorporating additional supportive components, such as online coaching technologies, could enhance the effectiveness of home-based exercise prehabilitation, warranting further investigation. Improvements in functional capacity achieved through exercise prehabilitation did not translate into reductions in overall complications or length of stay. Accordingly, future studies should explore other potential mechanisms—beyond functional capacity—that may mediate the effects of prehabilitation on clinical outcomes. Moreover, well-designed RCTs are needed that directly compare facility-based and home-based exercise prehabilitation under standardized intervention protocols with controlled external variables and should consider incorporating adherence metrics and exercise dose–response analyses to better control for exercise and multimodality.

## Data Availability

The original contributions presented in this study are included in the article/Supplementary Materials. Further inquiries can be directed to the corresponding author.

## Supplementary Materials

The following supporting information can be downloaded at: https://www.mdpi.com/article/10.3390/jcm15031274/s1, Table S1: PRISMA NMA Checklist; Table S2: Full search strategy for each database; Figure S1: Risk of Bias; Table S3: CINeMA Summary of Findings; Table S4: Global inconsistency results; Figure S2: Sensitivity analyses of 6MWD assuming different pre-post correlation coefficients.

## Abbreviations

The following abbreviations are used in this manuscript:

6MWD	6 min walk distance
RCT	Randomized controlled trial
SD	Standard deviation
LOS	Length of stay
NMA	Network meta-analysis
MD	Mean difference
RR	Risk ratio
CINeMA	Confidence in Network Meta-Analysis

## Appendix A

**Table A1:** Characteristics of included studies.

Study ID	Country	Cancer Type	Sample Size (n), Age (Mean)	Supervision Type	Exercise Protocol	Exercise Duration	Supervision	Timing of 6MWD Assessment	Comparator
Bousquet-Dion 2018 [37]	Canada	Colorectal	Prehab (n = 37) M = 30, F = 7 74 years Control (n = 26) M = 16, F = 10 71 years	Facility-based	30 min aerobic exercise, 3–4 days/week Resistance exercise, 3–4 times/week	4 weeks	Once weekly supervision by kinesiologist	4 weeks	Standard ERAS care
Chen 2024 [38]	China	Gastric	Prehab (n = 57) M = 33, F = 24 73 years Control (n = 58) M = 28, F = 30 74 years	Facility-based	30-min aerobic exercise, 3 days/week 3 sets resistance exercise, 2–3 times/week	3 weeks	All resistance exercise sessions supervised	4 weeks	Standard ERAS care
Danielsson 2025 [39]	Sweden	Colorectal	Prehab (n = 27) M = 11, F = 16 78 years Control (n = 25) M = 13, F = 12 77 years	Facility-based	Aerobic and Functional strength exercise, 5–6 times/week	2–3 weeks	At least 6 supervised training sessions lasting 1 h long	Discharge	Standard ERAS care
Fulop 2021 [40]	Hungary	Colorectal	Prehab (n = 77) M = 37, F = 40 70 years Control (n = 72) M = 39, F = 33 70 years	Facility-based	30 min aerobic and 10–15 min breath exercise daily	3–6 weeks	In-hospital supervised exercise, once per week	4 weeks	Standard ERAS care
Gillis 2025 [41]	Canada	Colorectal	Prehab (n = 51) M = 26, F = 25 59 years Control (n = 49) M = 24, F = 25 59 years	Home-based	Home-based walking, ≥150 min/week at moderate intensity Personalized functional exercise daily	2–3 weeks	Follow up via telephone once before and once after surgery	6 weeks	Standard ERAS care
Minnella 2018 [42]	Canada	Esophageal, Gastric	Prehab (n = 26) M = 18, F = 6 67.3 years Control (n = 25) M = 20, F = 5 68 years	Home-based	30-min aerobic exercise, 3 days/week 30 min resistance exercise, 1 day/week	4 weeks	Home-based, weekly telephone calls	4–8 weeks	Standard ERAS care
Molenaar 2023 [12]	Netherlands, Canada, Spain, Denmark, Italy	Colorectal	Prehab (n = 123) M = 62, F = 61 69 years Control (n = 128) M = 76, F = 52 71 years	Facility-based	4 × 2 min HIIT and resistance exercise, 3 times/week	4 weeks	Fully supervised exercise session	4 weeks	Standard ERAS care
Pesce 2024 [43]	Italy	Colon	Prehab (n = 35) M = 21, F = 14 68 years Control (n = 36) M = 21, F = 15 70 years	Facility-based	60 min aerobic exercise, 4 times/week Interval and resistance training, 3 times/week	4 weeks	3 supervised sessions per week included interval and resistance training	4 weeks	Standard ERAS care
Yang 2024 [44]	China	Colorectal	Prehab (n = 50) M = 34, F = 16 60 years Control (n = 45) M = 24, F = 21 64 years	Home-based	10–15 min light-intensity exercise, twice daily	2 weeks	WeChat or phone call daily	7 days	Standard ERAS care

ABBREVIATIONS. Prehab, Prehabilitation; 6MWD, 6-Minute Walk Distance; M, male; F, female; n, number of participants; min, minutes; ERAS, Enhanced Recovery After Surgery.
